# Acquisition of antibody isotypes against *Plasmodium falciparum* blood stage antigens in a birth cohort

**DOI:** 10.1111/j.1365-3024.2009.01165.x

**Published:** 2010-02

**Authors:** N O DUAH, D J C MILES, H C WHITTLE, D J CONWAY

**Affiliations:** 1 Department of Infectious and Tropical Diseases, London School of Hygiene and Tropical MedicineLondon, UK; 2 Medical Research Council LaboratoriesFajara, The Gambia; 3 South African Tuberculosis Vaccine Initiative, Institute of Infectious Diseases and Molecular Medicine, University of Cape TownCape Town, South Africa

**Keywords:** acquired immunity, antibody isotypes, birth cohort, blood stage antigens, Plasmodium falciparum

## Abstract

Information on the period during which infants lose their maternally derived antibodies to malaria and begin to acquire naturally their own immune responses against parasite antigens is crucial for understanding when malaria vaccines may be best administered. This study investigated the rates of decline and acquisition of serum antibody isotypes IgG1, IgG2, IgG3, IgG4, IgM and IgA to Plasmodium falciparum antigens apical membrane antigen (AMA1), merozoite surface proteins (MSP1-19, MSP2 and MSP3) in a birth cohort of 53 children living in an urban area in the Gambia, followed over the first 3 years of life (sampled at birth, 4, 9, 18 and 36 months). Antigen-specific maternally transferred antibody isotypes of all IgG subclasses were detected at birth and were almost totally depleted by 4 months of age. Acquisition of specific antibody isotypes to the antigens began with IgM, followed by IgG1 and IgA. Against the MSP2 antigen, IgG1 but not IgG3 responses were observed in the children, in contrast with the maternally derived antibodies to this antigen that were mostly IgG3. This confirms that IgG subclass responses to MSP2 are strongly dependent on age or previous malaria experience, polarized towards IgG1 early in life and to IgG3 in older exposed individuals.

## Introduction

Infants are protected from a number of infections by maternally transferred immunity. This immunity is not long-lasting, and children need to acquire immunity against common pathogens early in life. For malaria, a degree of resistance in endemic areas in the first 3–6 months of age has been attributed to a combination of protection by maternally transferred antibodies, foetal haemoglobin that cannot support parasite development, lack of p-aminobenzoic acid (PABA) in the infant breast milk diet which prevents parasite growth and limited exposure to vectors (1–7).

However, it has not been straightforward to demonstrate an association between maternal antibodies and protection of infants from malaria infection. A study conducted in Ghana showed that infants with high levels of maternal antibodies to *Plasmodium falciparum* antigens were found to be more susceptible to malarial attack, suggesting that maternally derived antibodies are an epidemiological marker for risk of infection in infants (2). A study in Tanzania also indicated that IgG antibodies to circumsporozoite protein (CSP) and merozoite surface protein 1 (MSP1) in infants were not protective against parasitaemia (8). Findings from a study of Nigerian infants also showed no association between levels of maternally derived antibodies reactive with parasite schizont extract and the age of first clinical episode of malaria (9). However, a study in Kenya showed that high levels of antibodies detected in maternal sera were associated with a longer time to first clinical malaria episode (10). From the varying results of these studies, the role of maternal antibodies in the protection of infants is unclear.

Positive serum IgG levels to several different *P. falciparum* blood stage antigens, such as merozoite surface proteins (MSP1-19, MSP1 block 2, MSP2), apical membrane antigen (AMA1) and erythrocyte binding antigen (EBA175), have been shown to be associated with reduced incidence of malaria in prospective cohort studies and where the subclass has been investigated, it is cytophilic and complement fixing IgG1 and IgG3 subclasses that are most strongly associated with protection (11–31). However, the IgG protective association was not seen with some of the antigens, such as MSP1-19 in Ghana, and also ring-infected erythrocyte surface antigen (RESA) and MSP1 in Papua New Guinea and Madagascar (17,32). IgG subclasses IgG2 and IgG4 have been found to lack the ability to activate cytotoxic cells and have been linked to the blocking of protective mechanisms (33). As the effect of antibodies is enhanced by their ability to bind Fc receptors on phagocytic cells, this can explain why IgG2 and IgG4 are not able to trigger phagocytosis, because the former binds to the FcRIIA-H131 (an allele of the Fc receptor that is not associated with protection) and the latter does not bind to FcRIIA at all. However, high IgG2 and low IgG4 levels have been associated with human resistance to *P. falciparum* malaria in Burkina Faso, where high level of IgG2 to RESA and merozoite surface protein 2 (MSP2) inversely correlated with risk of infection and *vice versa* for IgG4 (20). Similarly, in Cameroon high levels of IgG2 were associated with reduced risk of *P. falciparum* infection in infants from birth to 6 months of age (34). An *in vitro* study showed that IgG4 hinders IgG1- and IgG3-mediated opsonization of infected erythrocytes (35). There is thus some lack of consistent findings among protective association studies, which may be a result of parasite and host genetic variation as well as the design of the studies. Subclass specificities to blood stage antigens have been shown to display a level of age dependency. The predominant subclass for MSP1-19 antigen is IgG1 in all age groups, with the levels of both IgG1 and IgG3 increasing with age. For MSP2, there is age-related change from IgG1 to IgG3, whereas for AMA1, IgG1 is the predominant antibody and for MSP3, the predominant antibodies are IgG1 and IgG3 in all age groups (11,17,27,36–38).

The evidence indicating an association between age and the predominance of IgG1 and IgG3 responses to the *P. falciparum* MSP2 in endemic populations is quite strong. A study conducted in Tanzania with subjects from villages located at different altitudes (malaria transmission diminishes with altitude) demonstrated the effect of antigen, age and exposure on IgG subclass responses (39). The IgG1 subclass to AMA1 and MSP1-19 was predominant in sera from all age groups. However, IgG3 responses to MSP2 antigen were more age-dependent, with a predominance of IgG1 in younger children and of IgG3 in older children and adults. The IgG3 levels to MSP2 became more apparent after 2 years of age, and were higher than IgG1 by 15 years of age. This confirmed an earlier finding in the Gambia, where it was shown that IgG1 responses predominated in children but IgG3 to MSP2 antigens predominated in adults (11). Interestingly, a recent study on nonimmune travellers returning to France with *P. falciparum* malaria showed that their antibodies against MSP2 were not of the IgG3 subclass but were almost exclusively IgG1 (40). Therefore, it was hypothesized that differences exist in the rate of acquisition of different antibody isotypes or subclasses to blood stage malaria antigens, or that there are different determinants for these responses.

For the detection of differences in the rate of acquisition of antibodies to blood stage antigens, a birth cohort study is ideal. This study design involves the follow-up of infants over time as they may be exposed to natural infections, and allows for the estimation of the rate of acquisition of antibody isotypes over time in the individuals. Several vaccines that are under development are ultimately intended to prime responses to these antigens in infants, so naturally acquired responses need to be described adequately. This study investigated the rate of decline of maternally derived antibodies, and the acquisition of antibodies to *P. falciparum* blood stage antigens in a Gambian birth cohort, in an attempt to test for differences in the rates of decline and natural acquisition of antibody isotypes to blood stage antigens.

## Materials and Methods

### Study site and population

This study was performed in The Gambia where malaria transmission is highly seasonal, and most infections occur annually between August and November, during and immediately following the single rainy season from June to October (41). A cohort of 134 children recruited at birth (July 2003–February 2004) from the Sukuta Health Centre maternity ward was initially sampled to investigate antibody responses to alternative schedules of measles vaccination. The Health Centre serves an urban/peri-urban population that is part of the Greater Banjul Area in the coastal part of The Gambia. Infants from this area are mostly breast-fed into their second year of life. The recruitment criterion for the cohort was that births should be singletons with no congenital abnormalities, but with a birth weight of over 1·8 kg. The HIV status of the study subjects was unknown, but adult HIV prevalence in the region was below 2·5% at the time of the study (National AIDS Control Programme, unpublished data); therefore it was unlikely to be a significant confounder. All the subjects received childhood vaccinations as part of the Expanded Programme of Immunization (EPI) schedule, but were randomized to receive an extra dose of measles vaccine and were followed up from birth for the acquisition of immune responses. The children had access to the Health Centre, where a resident nurse was present to attend to them in case of any illness during the study period.

Cord blood samples and then blood samples at 4, 9, 18 and 36 months of age were collected, and the plasma stored at −20°C until antibody assays were performed. Of the 134 children recruited for the study on responses to measles vaccine, 53 had samples for all five time points up to the age of 36 months, and were therefore selected for the current investigation on naturally acquired antibodies to malaria antigens. Good data on post-natal episodes of malaria were not available because it was not part of the study design to capture these, and malaria is commonly treated presumptively in the community.

### *P. falciparum* blood stage antigens

In this study, the antigens employed in assays were *Escherichia coli* expressed recombinant proteins, as follows. The recombinant AMA1 is the full-length ectodomain representing amino acids 83–531 of AMA1 in the 3D7 clone (42). The 19-kDa fragment of MSP1-19 is a glutathione s-transferase (GST) fusion protein representing amino acids 1631–1726 of MSP1 in the Wellcome isolate (43). Two MSP2 allelic antigens are GST fusion proteins of MSP2 amino acid sequences 1–184 of the ch150/9 isolate and 22–247 of the Dd2 clone (44,45); Two MSP3 allelic antigens are maltose-binding protein (MBP) fusion proteins representing almost full-length sequences, amino acids 2–354 of the 3D7 clone and 2–379 of the K1 isolate (37). Recombinant proteins representing the GST and MBP fusion tags were also assayed as controls.

### Antibody assays

Enzyme-linked immunosorbent assays were used to detect the reactivity of antibody isotypes IgG1, IgG2, IgG3, IgG4, IgM and IgA in plasma to a panel of blood stage antigens AMA1, MSP1-19, MSP2-ch150/9, MSP2-Dd2, MSP3-3D7 and MSP3-K1. Briefly, wells of flat bottom 96-well plates (Immulon 4HBX, ThermoLabs, Waltham, MA, USA) were coated with 50 ng of recombinant antigen (all antigens) in 100 μL of coating buffer (15 mm Na_2_CO_3_, 35 mm NaHCO_3_, pH 9·3). The plates were incubated at 4°C overnight and then washed four times with PBS-T (PBS with 0·05% v/v Tween 20). Unoccupied protein binding sites on the plate were blocked with 200 μL per well of blocking buffer (1% skimmed milk, Marvel™ UK, in PBS-T) for 5 h at room temperature. Plates were washed prior to the addition of 100 μL of each plasma diluted with blocking buffer (1/500 for IgG1 and IgG3; 1/50 for IgG2, IgG4, IgM and IgA), dilutions chosen after optimization of assays for each isotype. After incubation at 4°C overnight, the plates were washed four times and 100 μL of horseradish peroxidase (HRP) conjugated sheep antibodies specific to detect each of the human IgG subclasses (The Binding Site, UK; anti-IgG1-4 reagents AP006-AP009), and HRP conjugated rabbit anti-human IgM or IgA (Dako, UK; reagents P0215 and P0216 respectively) at 1/5000 were added to each well and incubated at room temperature for 3 h. Plates were washed four times and antibody reactivities detected with 100 μL of substrate (0·4 mg/mL of *o*-phenylenediamine; 0·08% v/v H_2_O_2_) in developing buffer (24·5 mm citric acid monohydrate; 52 mm Na_2_HPO_4_, pH 5·0). The reaction was stopped with 50 μL of 2 m H_2_SO_4_ and optical density (OD) of plates measured at 490 nm. The assays were run in duplicate wells with different time point samples from each individual run on the same plate. The OD reading was corrected for nonspecific binding by subtracting the OD for antibody reactivity to the relevant fusion tags, MBP for MSP3 recombinant proteins and GST for MSP1 and MSP2 recombinant proteins. Antibody isotype reactivities of 20 nonimmune negative control individuals (European tourists in The Gambia) were also assayed.

### Data analysis

Data generated from assays in the form of ELISA OD values were entered into Microsoft Excel worksheets and then transferred to SPSS version 11 (SPSS Inc., Chicago, IL, USA) and GraphPad Prism version 4·0 (GraphPad Software, San Diego, CA, USA) for analysis. An antibody isotype level was considered positive if the individual’s OD reading was above a cut-off value (mean + 3 standard deviation of values from the panel of 20 nonimmune control sera). Statistical comparisons of antibody isotype levels in cord blood among different calendar birth months were performed using Wilcoxon’s signed rank test.

## Results

### Effect of month of birth on antibody isotype levels in cord blood sera

**Figure 1:**
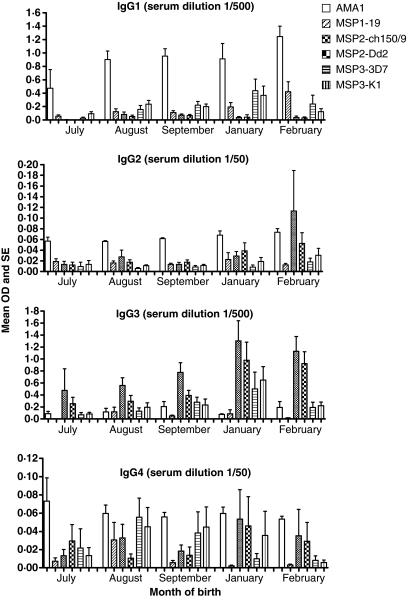
The mean OD and SE of cord blood plasma antibody isotype reactivities to antigens for children in each birth month (July, *n* = 5; August, *n* = 15; September, *n* = 17; January, *n* = 7; February, *n* = 9). Serum dilutions used for the detection of IgG subclasses are indicated in brackets in the graph (1/500 for IgG1 and IgG3, 1/50 for IgG2 and IgG4).

Of the 53 children analysed with all time point samples in the cohort, 5 (10%) were born in July 2003, 15 (28%) in August 2003, 17 (32%) in September 2003, 7 (13%) in January 2004 and 9 (17%) in February 2004. The mean levels of cord blood antibodies to the different antigens for each birth month are shown in Figure 1. There were differences in the levels of IgG1 and IgG3 to most antigens between the birth months, with levels generally increasing after July 2003. Considering the profile of reactivities to the panel of six antigens for each isotype, comparisons were made between levels in the cord blood in different birth months. Testing all pairwise comparisons separately, there were significantly lower IgG1 levels in cord blood of births in July 2003 than that in August 2003 (*P* = 0·04) or January 2004 (*P* = 0·02). For IgG3, levels were significantly lower in July 2003 than in August 2003 (*P* = 0·005), September 2003 (*P* = 0·006) or January 2004 (*P* = 0·02), and levels were also lower in August 2003 than in January 2004 (*P* = 0·04). Levels of IgG2 were very low generally, although even lower in September 2003 than in January 2004 (*P* = 0·01). For IgG4, there were no differences in levels between the birth months. Most of these differences were not significant after a Bonferroni correction for multiple comparisons, except for IgG3 levels that were significantly lower in July 2003 (prior to the malaria season) than that in other months.

### Proportion of children with positive antibody reactivities to blood stage antigens in cord blood

The relative levels of different antibody isotypes in cord blood sera were observed to vary among antigens (Figure 1). Prevalence of IgG1 positivity was highest for AMA1 (94%), followed by MSP3-3D7 and MSP3-K1 (66% each), MSP1-19 (62%), MSP2-Dd2 (28%) and MSP2-ch150/9 (25%). In contrast, IgG3 positivity was highest to MSP2-Dd2 and MSP2-ch150/9 (85% and 83% respectively), followed by MSP3-K1 and MSP3-3D7 (53% and 43% respectively), AMA1 (32%) and MSP1-19 (26%). For IgG2 and IgG4, proportions positive were highest to MSP2-ch150/9 (53% and 45% respectively). No positive reactivities were observed for IgM and IgA to any of the antigens in the cord sera, consistent with the suggestion that these two isotypes are not transferred through the placenta to infants by mothers.

### Decline of antibody isotype responses to blood stage antigens

**Figure 2:**
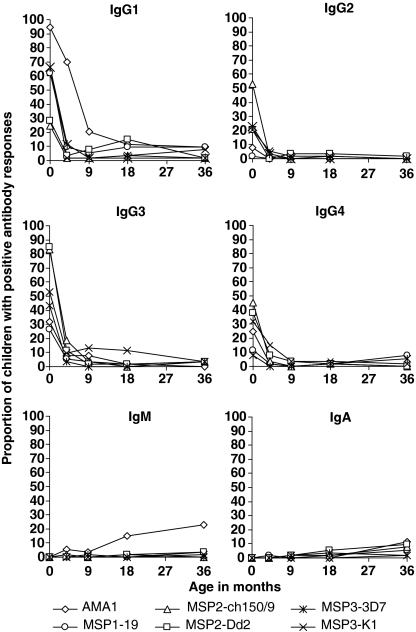
Proportion of children with positive reactivities of antibody isotypes IgG1, IgG2, IgG3, IgG4, IgM and IgA to antigens at ages 0, 4, 9, 18 and 36 months. Each line represents the change in proportions of children with positive reactivities (ELISA OD >mean + 3 SD of negative control sera panel) against specific antigens at different ages.

Antibody isotype reactivities to malaria antigens were analysed for samples collected at follow-up ages 4, 8 or 9, 18 and 36 months. A decline of all the IgG subclasses to all antigens was first observed in the children at 4 months of age. Detectable IgG1 to AMA1 persisted for longer because of high initial levels, as demonstrated by the proportion of children with positive levels at 4 months of age (Figure 2). After 9 months, most antibody levels were undetectable, except IgG1 to AMA1 in a minority of individuals.

### Acquisition of antibody isotypes

**Figure 3:**
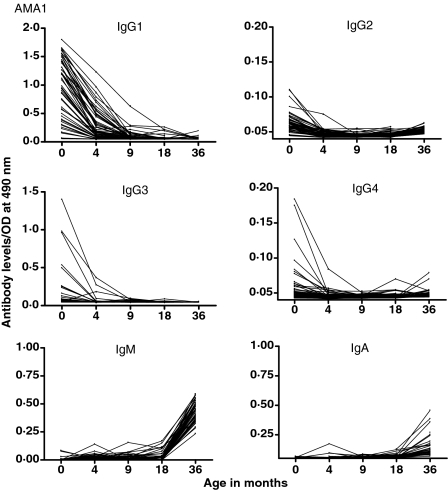
The decline and acquisition of the antibody isotypes, IgG1, IgG2, IgG3, IgG4, IgM and IgA, to AMA1 over the five time points 0, 4, 9, 18 and 36 months. Each line represents the measurement of antibody levels in an individual at different ages.

**Figure 4:**
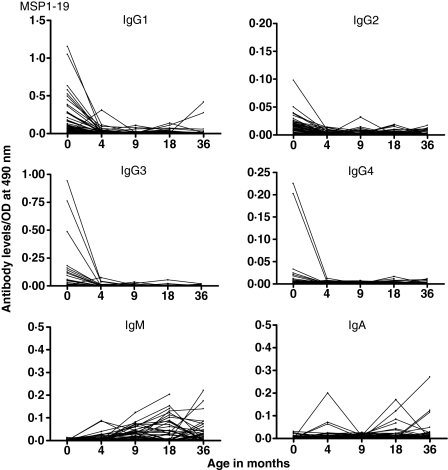
The decline and acquisition of the antibody isotypes, IgG1, IgG2, IgG3, IgG4, IgM and IgA, to MSP1-19 over the five time points 0, 4, 9, 18 and 36 months. Each line represents the measurement of antibody levels in an individual at different ages.

**Figure 5:**
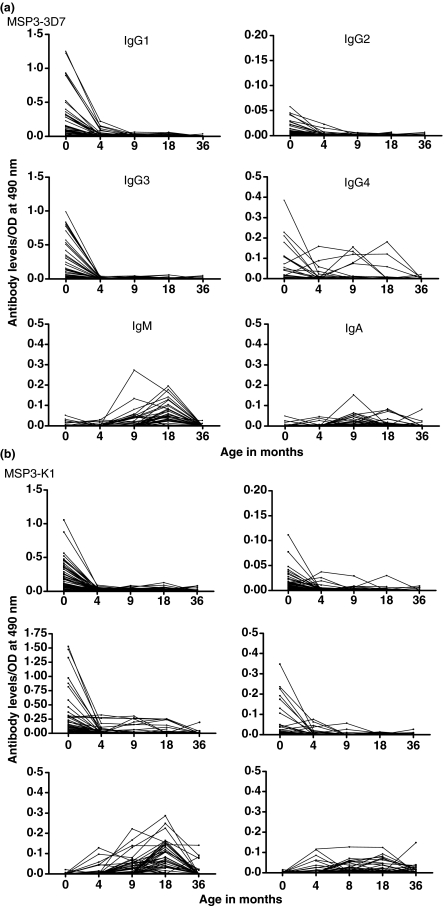
(a) The decline and acquisition of the antibody isotypes, IgG1, IgG2, IgG3, IgG4, IgM and IgA, to MSP3-3D7 over the five time points 0, 4, 9, 18 and 36 months. Each line represents the measurement of antibody levels in an individual at different ages. (b) The decline and acquisition of the antibody isotypes, IgG1, IgG2, IgG3, IgG4, IgM and IgA, to MSP3-K1 over the five time points 0, 4, 9, 18 and 36 months. Each line represents the measurement of antibody levels in an individual at different ages.

The primary antibody isotype IgM to AMA1 was detected in some children, with positive responses increasing with age, although only in a minority of individuals, 6% at 4 months, 15% at 18 months and 23% at 36 months (Figure 2). To each of the antigens, IgA was detected in a small minority, increasing after 9 months of age. Acquisition of IgG1 to AMA1, MSP1-19 and MSP2 occurred in a few individuals from 4 or 9 months onwards, and to MSP3 from 18 months of age. IgG1 to MSP1-19 was acquired by 9% of the children from 18 to 36 months. For the MSP2 allelic antigens, IgG1 to MSP2-ch150/9 was acquired by 4% of the children at 18 months, while 8% and 15% of the children were positive for responses against MSP2-Dd2 at 9 and 18 months respectively. IgG1 response against MSP3-K1 was also found in 4% and 8% of the children at 18 and 36 months, respectively. Figures 3, 4, 5a, b shows the decline and acquisition of the antibody isotypes IgG1, IgG2, IgG3, IgG4, IgM and IgA to AMA1, MSP1-19, MSP3-3D7 and MSP3-K1, respectively.

**Figure 6:**
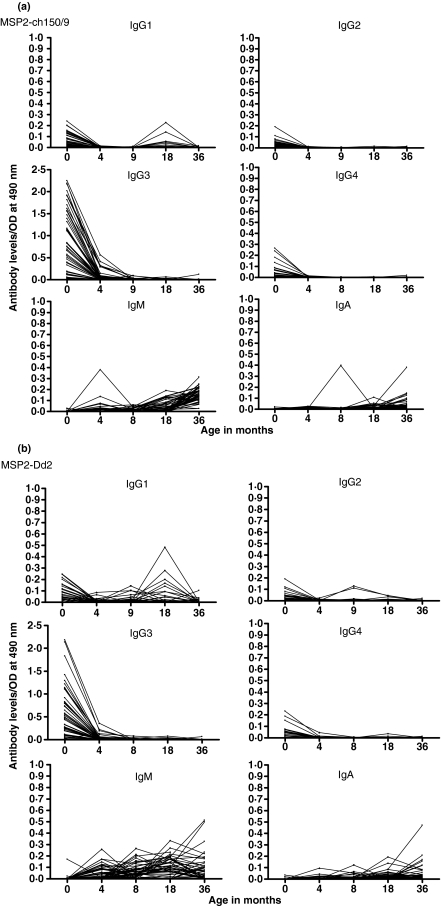
(a) The decline and acquisition of antibody isotypes IgG1, IgG2, IgG3, IgG4, IgM and IgA to MSP2-ch150/9 at the ages 0, 4, 9, 18 and 36 months. Each line represents the measurement of antibody levels for an individual at different ages. (b) The decline and acquisition of the antibody isotypes, IgG1, IgG2, IgG3, IgG4, IgM and IgA, to MSP2-Dd2 over the five time points 0, 4, 9, 18 and 36 months. Each line represents the measurement of antibody levels in an individual at different ages.

Maternally transferred IgG subclass reactivity to MSP2 in the cord blood was dominated by IgG3. In contrast, acquisition of IgG1 to MSP2 occurred in individual children (Figure 6a, b), whereas there was no observation of an IgG3 response to MSP2 in any of the children.

## Discussion

Maternally transferred antibody reactivities in cord blood to the panel of antigens followed the polarized pattern of IgG1 to AMA1 and MSP1-19, and IgG3 for MSP2-ch150/9 and MSP2-Dd2, previously described for endemic sera in The Gambia and elsewhere (11,39,44,45). A more equal distribution of both IgG1 and IgG3 isotypes against MSP3 was observed, as reported previously (37,46). The decline of maternally derived IgG subclass antibodies occurred as expected, so that by 4 months, most of the antibodies were depleted.

The birth month of the children was associated with differences in the level of antibodies detected in the cord plasma. The Gambia experiences annual rainfall from June to October and thus seasonal malaria transmission occurs at this period, with a peak of incidence between August and November (41,47). Therefore, it was not surprising to observe relatively low antibody isotype levels in the cord plasma from children born in July. Significantly higher levels of antibody isotypes to the antigens were seen in children born from August 2003 to February 2004, implying a boost of antibody responses in some of the mothers because of exposure to malaria parasites during the transmission season. Antibodies previously acquired would to some extent be depleted during the dry season as a result of lack of boosting by re-infection (48), and consequently lower levels of antibodies would be transferred by mothers in July (2,9,10).

It is important to note that although antibodies to MSP2 are predominantly IgG3 in the cord blood, children who made a response produced IgG1 against MSP2. This shows that IgG1 is the first IgG subclass synthesized against this malaria antigen after primary infections. This is consistent with the previous suggestion that there is an exposure-related shift from IgG1 to IgG3 to MSP2 antigens (11,49). It also implies that IgG3 is produced in the later years, probably in response to subsequent infections. As isotype subclass switching cannot occur in B-cell lines from IgG1 to IgG3, the IgG3 response to MSP2 antigen in latter years or subsequent infections will rely on the formation of new B cells with an IgM switch to IgG3. Adults living in malaria endemic areas have high levels of cytophilic antibodies IgG1 and IgG3 to malaria antigens and these may be protective. It is possible that the IgG1 responses in young children (rather than IgG3) may not be protective, but that IgG3 responses that are acquired later are more protective (31).

An increase of antibody levels in these children was expected with age. However, it was observed that the proportions with any antibody responses from 9 months of age onwards were very low. A recent decline in the incidence of malaria in The Gambia, particularly since 2003 (41), accounts for a low level of exposure in this cohort. A majority of the children sleep under insecticide-treated bed nets and are therefore protected from infectious bites at night. It has sometimes been considered important that children in endemic areas have some exposure to malaria parasites, to acquire immunity naturally, as otherwise they might be more likely to experience severe disease in the event of a future epidemic of malaria. Therefore, further studies of the immunological and clinical consequences of declining malaria endemicity are needed.
